# Spatio-temporal variability of trace elements fingerprints in cockle (*Cerastoderma edule*) shells and its relevance for tracing geographic origin

**DOI:** 10.1038/s41598-017-03381-w

**Published:** 2017-06-14

**Authors:** Fernando Ricardo, Tânia Pimentel, Luciana Génio, Ricardo Calado

**Affiliations:** Departamento de Biologia & CESAM, Universidade de Aveiro, Campus Universitário de Santiago, 3810-193 Aveiro, Portugal

## Abstract

Understanding spatio-temporal variability of trace elements fingerprints (TEF) in bivalve shells is paramount to determine the discrimination power of this analytical approach and secure traceability along supply chains. Spatio-temporal variability of TEF was assessed in cockle (*Cerastoderma edule*) shells using inductively coupled plasma-mass spectrometry (ICP-MS). Four elemental ratios (Mg/Ca, Mn/Ca, Sr/Ca and Ba/Ca) were measured from the shells of specimens originating from eight different ecosystems along the Portuguese coast, as well as from four different areas, within one of them, over two consecutive years (2013 and 2014). TEF varied significantly in the shells of bivalves originating from the eight ecosystems surveyed in the present study. Linear discriminant function analyses assigned sampled cockles to each of the eight ecosystems with an average accuracy of 90%. Elemental ratios also displayed significant differences between the two consecutive years in the four areas monitored in the same ecosystem. Overall, while TEF displayed by cockle shells can be successfully used to trace their geographic origin, a periodical verification of TEF (>6 months and <1 year) is required to control for temporal variability whenever comparing specimens originating from the same area collected more than six months apart.

## Introduction

The common cockle (*Cerastoderma edule*) is one of the most abundant bivalves in the estuaries and bays of the European Atlantic coastline^1–3^ and supports several commercially important fisheries^2, 4^. Considering their distribution, sedentary lifestyle and feeding behavior (filtration), cockles can be considered as highly prone to bioaccumulation of pollutants and a wide range of pathogenic microorganisms (e.g. *Escherichia coli*, *Salmonella* spp., *Vibrio* spp., norovirus)^5–9^. The traditional way of consuming bivalves, either raw or undercooked, increases the risks of transmitting infection to humans^10^ and thus makes these organisms a high-risk group to seafood industry players and health agencies^11^. To protect consumers from potential risks, the European Union (EU) classifies the capture/production areas of bivalves according to the loads of *E*. *coli* present in the flesh and intra-valvular liquid of live specimens^12, 13^. In this way, it is paramount in terms of seafood safety to know as accurately as possible the geographic origin of bivalves being traded for human consumption (e.g. adjacent areas, even in the same ecosystem, may display contrasting classifications for their capture/production).

Nowadays, in an era of seafood trade globalization, the technological developments in food production, handling, processing and distribution allow to increase the distance travelled by seafood from producers to consumers^14–17^. To cope with this new paradigm, the European regulation (EC) No 1379/2013^18^ requires labels to display the scientific name of the species being traded, the production method (captured or produced) and geographical area of capture/production. Currently, for bivalves, detailed information on harvesting areas is not provided. In this context, this omission paves the way for potential commercial fraud and jeopardizes efforts to enhance seafood safety (e.g. the illegal commercialization of bivalves originating from areas where their capture/production is forbidden).

The implementation of biotechnological tools for the authentication of geographic origin of seafood products can therefore play a pivotal role towards a safer globalized trade^14, 19^. However, most efforts so far have tried to address issues related with species mislabelling rather than with the traceability of geographical origin. Trace element fingerprints (TEF) of bivalve mineral structures (e.g. shells) have been successfully used as natural tags to discriminate specimens from different geographical origin^20, 21^. TEF variability in the hard mineral structures (e.g. shells) of marine bivalves is influenced by the availability of trace elements in seawater, which reflect shifts in the environmental features of the ecosystem(s) occupied by these organisms^22, 23^. Being inert structures, TEF displayed by bivalve shells are chronological deposited fingerprints that reflect the geographical surroundings of a given specimen from birth to its harvest^24–27^. These unique “natural tags” can be analysed through inductively coupled plasma-mass spectrometry (ICP-MS)^20^. A wide range of element/calcium ratios can be measured in these calcified structures, the most common ones being Ba/Ca, Cd/Ca, Cu/Ca, Cr/Ca, Mg/Ca, Mn/Ca, Pb/Ca, Sr/Ca, U/Ca and Zn/Ca^20, 26, 28^. The discrimination of specimens from geographically close populations or stocks (20–50 km)^29, 30^, as well as from adjacent bivalve capture/production areas (<1 km apart)^20^, has been successfully achieved using TEF of bivalve shells. However, in order to be able to use this approach to confirm the geographic origin claimed by producers and/or traders, thus securing traceability along seafood supply chains, it is important to put it to the test in realistic case study scenarios. Moreover, it is also necessary to determine the temporal stability of TEF in adult bivalves and consider how any potential variability may bias the discrimination of the geographic origin of target specimens. The potential existence of temporal variability in TEF may be highly relevant for agencies willing to use this approach to expose fraudulent practices and take legal action.

**Figure 1:**
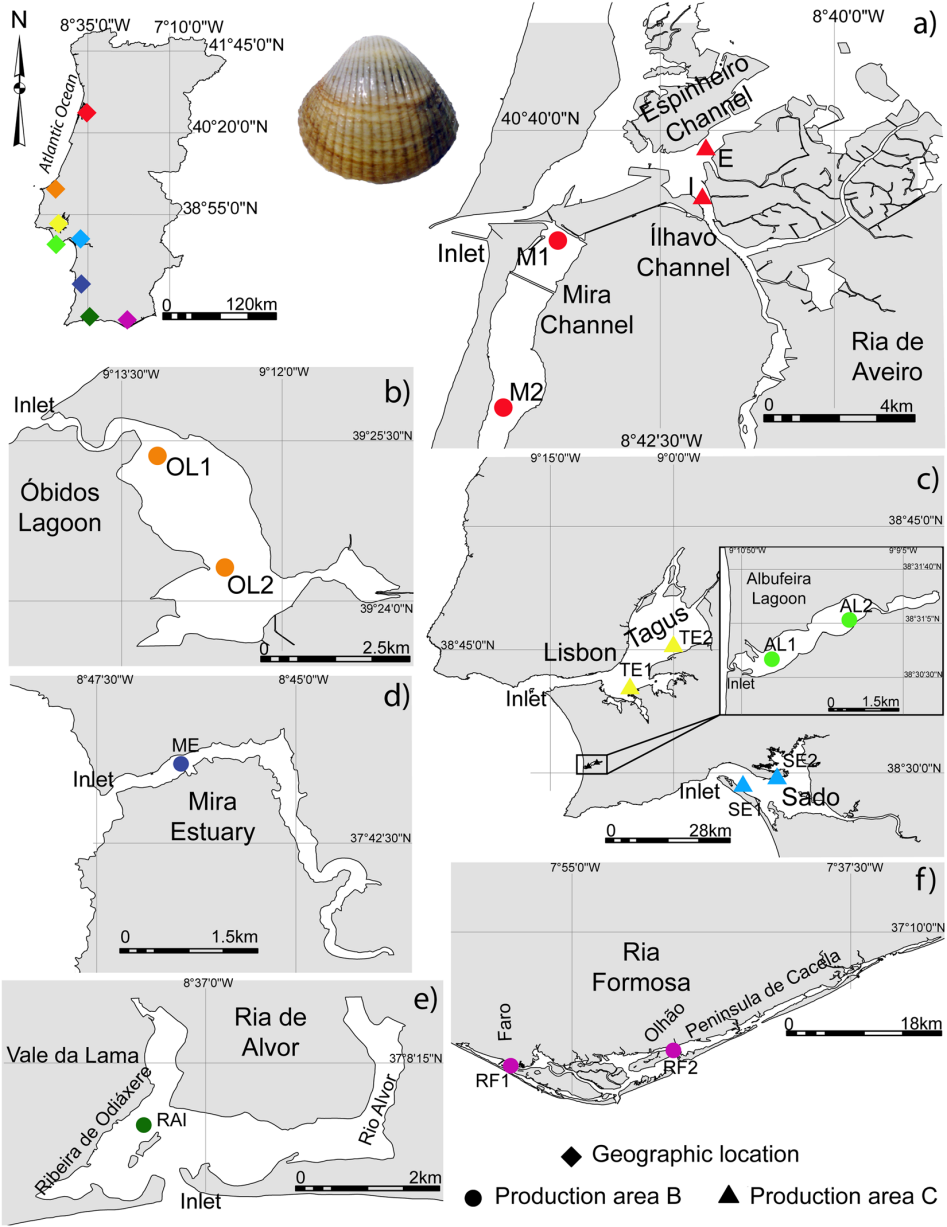
Sampling locations of *Cerastoderma edule* in mainland Portugal: (**a**) Ria de Aveiro (RAv; M1:40°38′26.30″N, 8°43′58.90″W; M2: 40°35′58.30″N, 8°44′47.80″W; I: 40°38′35.40″N, 8°41′35.40″W and E: 40°39′′48.50″N, 8°41′45.03″W), (**b**) Óbidos lagoon (OL1: 39°25′20.34″N, 9°13′14.54″W and OL2: 39°24′2.01″N, 9°12′30.91″W), (**c**) Tagus estuary (TE1: 38°39′27.44″N, 9°6′35.95″W and TE2: 38°44′5.18″N, 9°0′46.54″W), Albufeira lagoon (AL1: 38°30′36.67″N, 9°10′32.96″W and AL2: 38°31′1.33″N, 9°9′53.16″W) and Sado estuary (SE1: 38°27′46.00″N, 8°51′32.00″W and SE2: 38°29′13.25″N, 8°48'52.79″W) (**d**) Mira estuary (ME: 37°43′30.60″N, 8°46′15.40″W), (**e**) Ria de Alvor (RAl: 37°07′55.7″N, 8°37′27.40″W) and (**f**) Ria Formosa (RF1: 37°00'23.20″N, 7°59′28.40″W and 37°01'24.30″N, 7°49′49.50″W). The map was created using the software ArcGIS v10.2.2.

The present study aimed to evaluate if TEF of cockle shells from specimens captured in eight different ecosystems along the Portuguese Atlantic coastline (Fig. 1), where commercial fisheries targeting the live trade of this species occurs, can be used to successfully discriminate their geographic origin. The present study also aimed to determine the temporal stability of TEF in cockle shells between two consecutive years (2013 and 2014) in areas within the same ecosystem (Fig. 1a) but displaying different classifications (according to European regulation (EC) No 1379/2013^18^ for the capture/production of bivalves.

## Results

**Figure 2:**
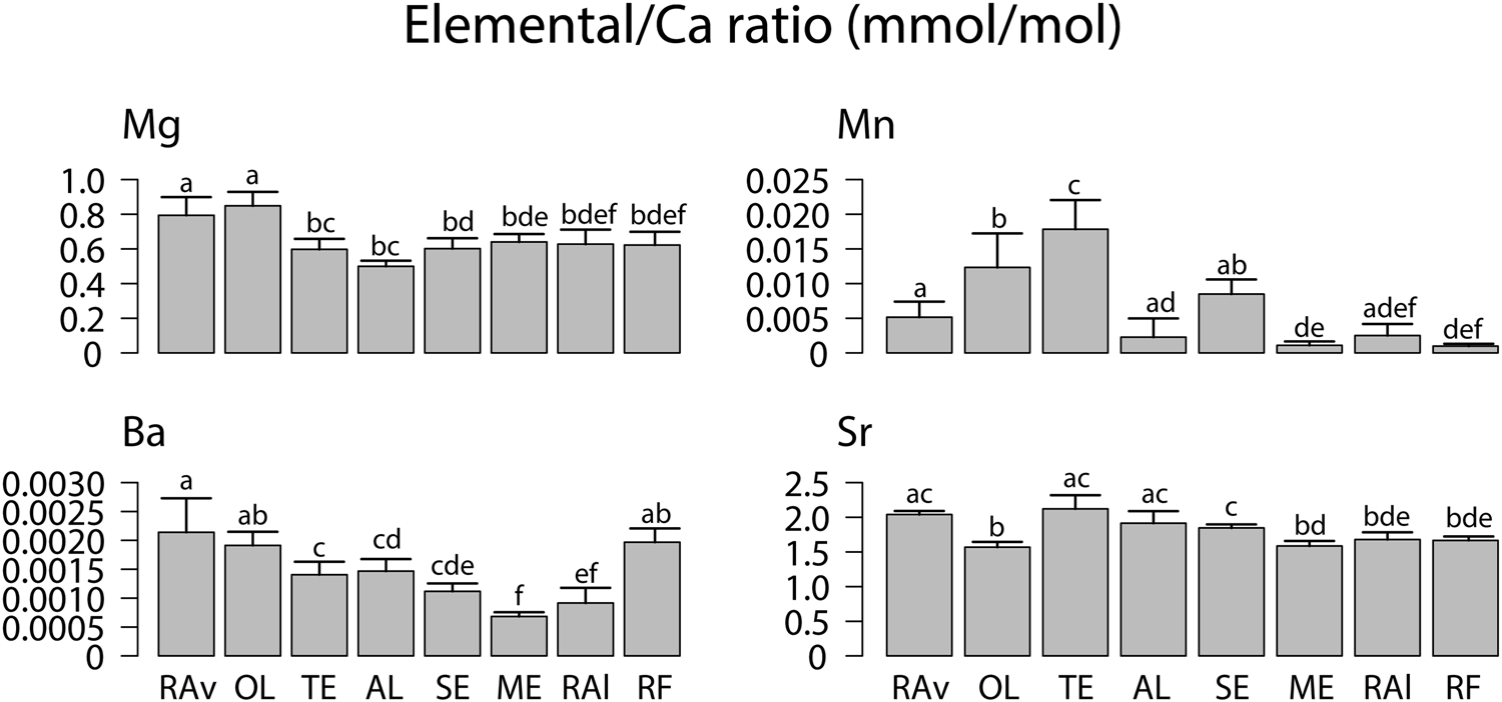
Ratios of trace elements to Calcium (Ca) concentrations (mmol to mol) (average ± SD; n = 10) of *Cerastoderma edule* shells from eight ecosystems along the Portuguese coast: Ria de Aveiro (RAv), Óbidos lagoon (OL), Tagus estuary (TE), Albufeira lagoon (AL), Sado estuary (SE), Mira estuary (ME), Ria de Alvor (RAl) and Ria Formosa (RF). Different letters on different ecosystems represent the existence of significant differences for each trace element ratio (*p* < 0.05).

Trace element fingerprints (TEF) of *Cerastoderma edule* shells differed among ecosystems, with the exception of Mira estuary (ME) and Ria de Alvor (RAl), with MANOVA analyses revealing strong significant differences when considered all trace elements together (Table 1). Considering each element separately, cockles from Ria de Aveiro (RAv) and Óbidos lagoon (OL) registered the highest Mg/Ca and were not significantly different from each other (*p* ≥ 0.05), but differed significantly from specimens originating from other ecosystems (*p* < 0.05) (Fig. 2). Cockles from the Tagus estuary (TE) showed the highest Mn/Ca with significant differences being recorded between specimens from this ecosystem and conspecifics collected in all others ecosystems that were surveyed (*p* < 0.05) (Fig. 2). Ba/Ca was higher in cockle shells from RAv, OL and Ria Formosa (RF), with significant differences being recorded when compared with that of cockles from all other ecosystems (*p* < 0.05) (Fig. 2). Concerning Sr/Ca, all specimens collected in the ecosystems surveyed in this study displayed a similar ratio for this element, although significant differences (*p* < 0.05) were still recorded between *C*. *edule* shells from different ecosystems (Fig. 2).

**Table 1:** Multivariate analysis of variance (MANOVA) of trace elements fingerprints of *Cerastoderma edule* shells from eight ecosystems along the Portuguese coast: Ria de Aveiro (RAv), Óbidos lagoon (OL), Tagus estuary (TE), Albufeira lagoon (AL), Sado estuary (SE), Mira estuary (ME), Ria de Alvor (RAl) and Ria Formosa (RF).

Ecosystem	df	Pillai	approx. F	*p*	Bonferroni *p*. adjust
RAv vs OL	1	0.948	67.82	0.000	0.000
RAv vs TE	1	0.946	66.20	0.000	0.000
RAv vs AL	1	0.938	56.85	0.000	0.000
RAv vs SE	1	0.943	62.61	0.000	0.000
RAv vs ME	1	0.975	147.77	0.000	0.000
RAv vs RAl	1	0.938	56.84	0.000	0.000
RAv vs RF	1	0.981	194.73	0.000	0.000
OL vs TE	1	0.948	68.35	0.000	0.000
OL vs AL	1	0.950	71.95	0.000	0.000
OL vs SE	1	0.969	117.21	0.000	0.000
OL vs ME	1	0.977	158.33	0.000	0.000
OL vs RAl	1	0.945	63.95	0.000	0.000
OL vs RF	1	0.881	27.74	0.000	0.000
TE vs AL	1	0.908	36.99	0.000	0.000
TE vs SE	1	0.699	8.73	0.001	0.021
TE vs ME	1	0.919	42.28	0.000	0.000
TE vs RAl	1	0.892	31.12	0.000	0.000
TE vs RF	1	0.954	77.94	0.000	0.000
AL vs SE	1	0.838	19.42	0.000	0.000
AL vs ME	1	0.936	55.20	0.000	0.000
AL vs RAl	1	0.720	9.63	0.000	0.013
AL vs RF	1	0.848	20.86	0.000	0.000
SE vs ME	1	0.935	54.02	0.000	0.000
SE vs RAl	1	0.821	17.14	0.000	0.001
SE vs RF	1	0.945	64.73	0.000	0.000
ME vs RAl	1	0.531	4.24	0.017	0.480
ME vs RF	1	0.940	59.14	0.000	0.000
RAl vs RF	1	0.891	30.68	0.000	0.000

**Figure 3:**
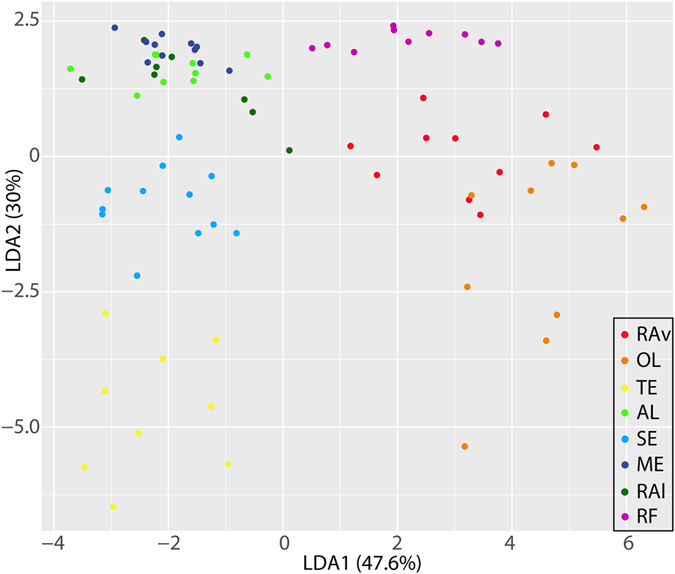
Linear discriminant analysis (LDA) of cockles based on trace elements fingerprints of shells collected from eight different ecosystems along the Portuguese coast: Ria de Aveiro (RAv), Óbidos lagoon (OL), Tagus estuary (TE), Albufeira lagoon (AL), Sado estuary (SE), Mira estuary (ME), Ria de Alvor (RAl) and Ria Formosa (RF).

The first two discriminant functions of the linear discriminant analysis (LDA) explained 77.6% of the TEF variation in the data set (LDA 1: 47.6% and LDA 2: 30%) (Fig. 3). Specimens collected in RAv, RF, OL and Sado estuary (SE) displayed the highest percentages of correct classification (100%), whereas for cockles originating from TE and ME a single specimen was misclassified (thus resulting, in 90% of correct classifications). Most misclassifications were associated with *C*. *edule* collected in Albufeira lagoon (AL) and RAl, with respectively 20 and 40% of the specimens collected being erroneously assigned to other ecosystems (Table 2).

**Table 2:** Classification success (by ecosystem) of a linear discriminant analysis (LDA) for *Cerastoderma edule* shells based on trace element fingerprints. Ria de Aveiro (RAv), Óbidos lagoon (OL), Tagus estuary (TE), Albufeira lagoon (AL), Sado estuary (SE), Mira estuary (ME), Ria de Alvor (RAl) and Ria Formosa (RF).

Original Ecosystem	% Predicted Ecosystem								Total per ecosystem	% Correct (ecosystem)
	RAv	OL	TE	AL	SE	ME	RAl	RF		
RAv	100	0	0	0	0	0	0	0	10	100
Original Ecosystem	0	100	0	0	0	0	0	0	10	100
TE	0	0	90	0	10	0	0	0	10	90
AL	0	0	0	80	10	0	10	0	10	80
SE	0	0	0	0	10	0	0	0	10	100
ME	0	0	0	0	0	90	10	0	10	90
RAl	0	0	0	20	0	20	60	0	10	60
RF	0	0	0	0	0	0	0	100	10	100
Average classification success										90

.

**Figure 4:**
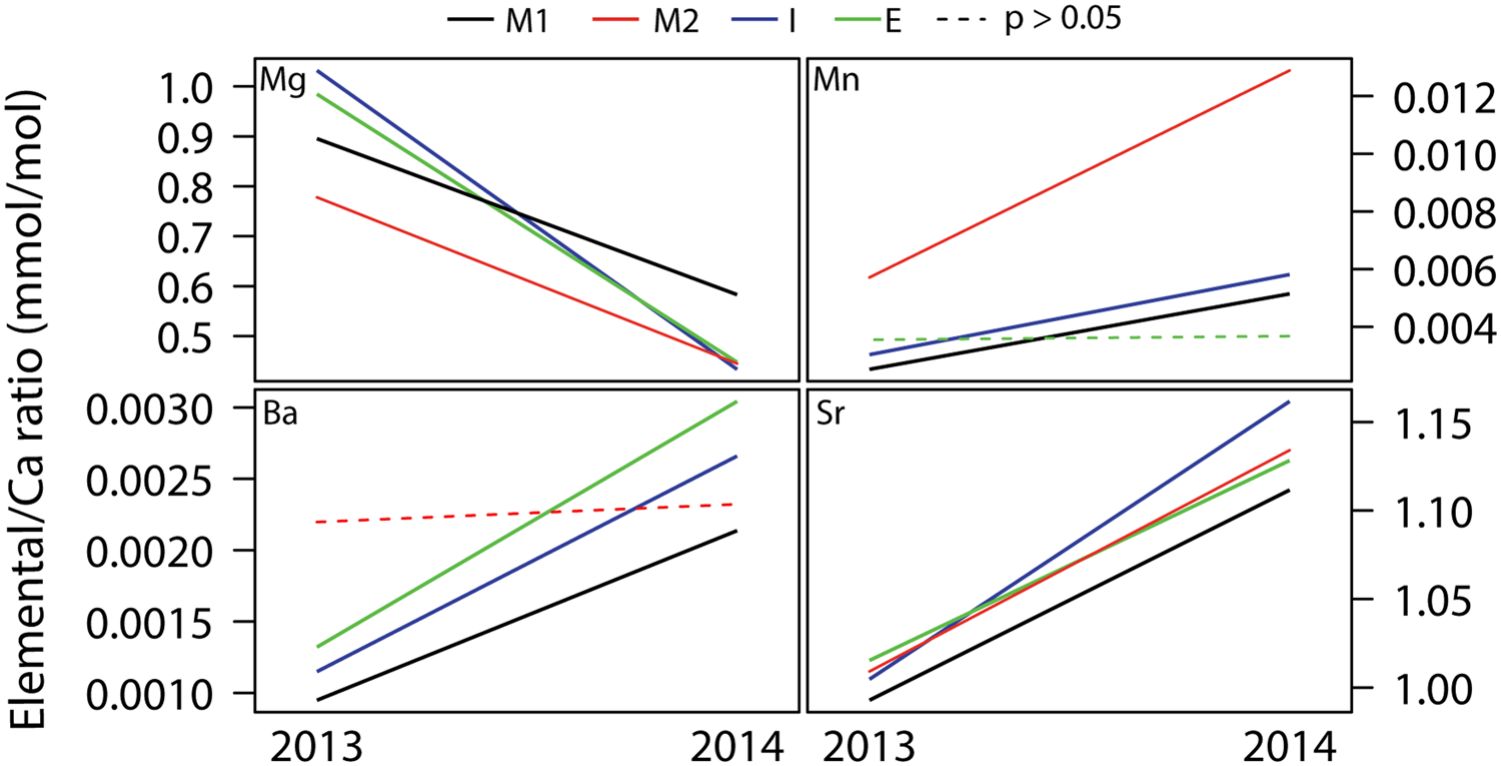
Evolution of elemental ratios of Mg, Mn, Ba and Sr in trace elements fingerprints (TEF) of cockle shells from 2013 to 2014 in areas: Mira Channel (M1 and M2), Ílhavo Channel (I) and Espinheiro Channel (E). The dotted lines represent significant differences in the elemental ratio between years (*p* < 0.05).

**Figure 5:**
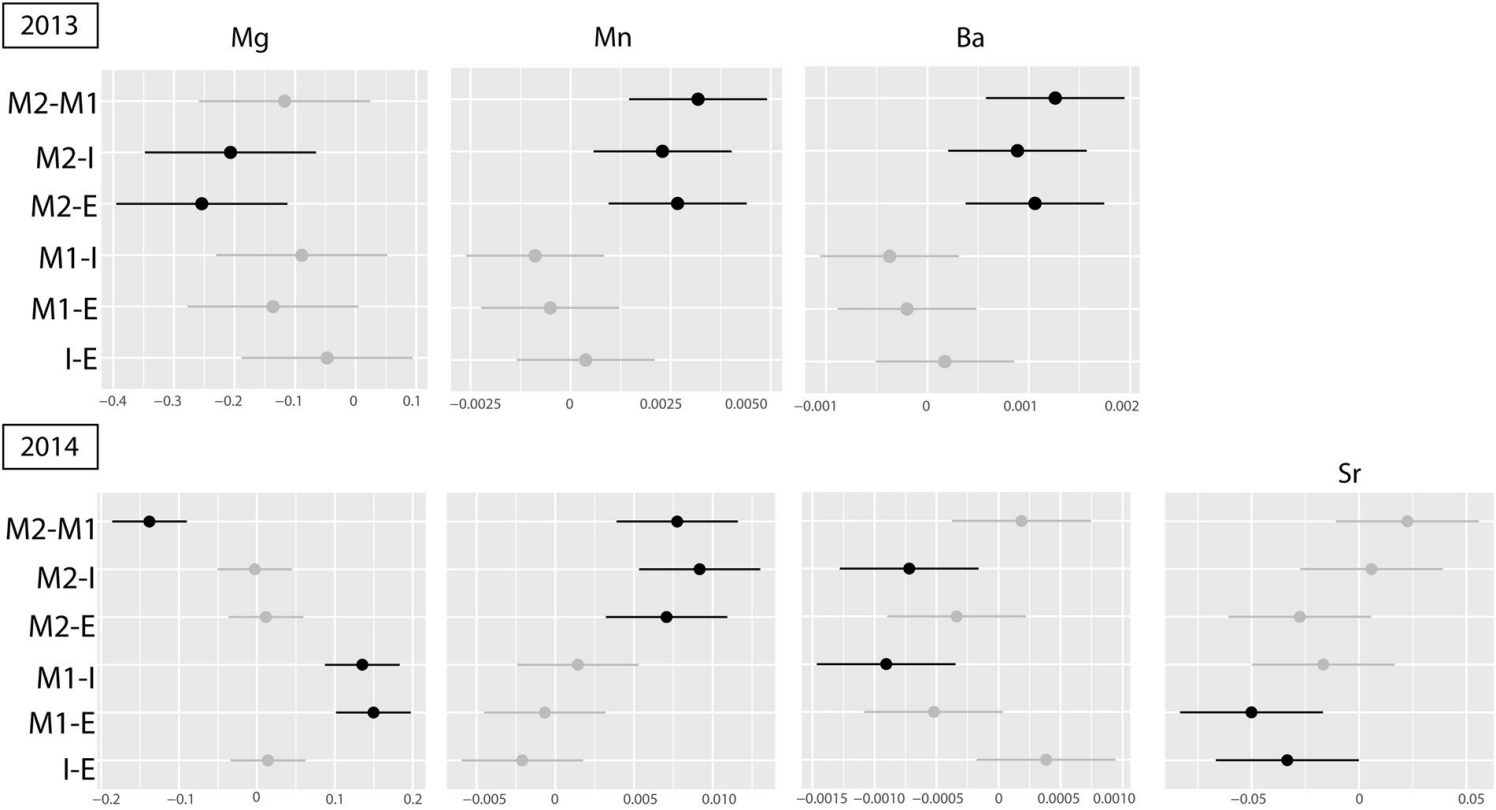
Tukey plot with black lines indicating significant differences in elemental ratios of trace elements fingerprints in cockle shells among areas within Ria de Aveiro (ANOVA, Tukey test comparisons, *p* < 0.05) over two consecutively years (2013 and 2014).

In RAv, TEF of *C*. *edule* shells showed that, with the exception of Mg/Ca, all ratios (Mn/Ca, Ba/Ca and Sr/Ca) increased from 2013 to 2014 (Fig. 4). As a significant interaction between time x areas was recorded for all ratios (MANOVA, F = 8.24, *p* < 0.0001) and significant differences between areas were also recorded for each year (MANOVA, F = 9.44, *p* < 0.0001), the analysis of TEF of *C*. *edule* shells was made separately for each area to avoid the masking of any potential inter-annual differences. This analysis revealed that Mg/Ca, Mn/Ca, Ba/Ca and Sr/Ca, varied significantly between 2013 and 2014 in all areas, with exception of areas E and M2 for Mn/Ca and Ba/Ca, respectively (Fig. 4). TEF of *C*. *edule* shells was analysed between areas for each year. In 2013, Ba/Ca, Mn/Ca and Mg/Ca showed significant differences between area M2 and all other areas, except M2 and M1 for Mg/Ca (Fig. 5). In 2014, Mg/Ca was the sole element that revealed significant differences between area M1 and all other areas. The ratio of Ba/Ca was significantly different between area I and areas M1 and M2, whereas the Sr/Ca was significantly different between area E and areas M1 and I (Fig. 5).

## Discussion

Trace element fingerprints (TEF) displayed by cockle shells exhibited significant differences among ecosystems, most likely due to the prevalence of highly dynamic biogeochemical processes such as (e.g. in estuaries and coastal lagoons). The recurrent shifting of environmental conditions (e.g. salinity, temperature, rainfall) in these ecosystems promotes more or less dramatic changes in water chemistry, which are ultimately reflected in the TEF^31, 32^ of specimens colonizing these habitats. Indeed, the ratios of the trace elements monitored in the present study (Mg/Ca, Mn/Ca, Sr/Ca and Ba/Ca) have already been reported to display significant spatio-temporal variations in the shells of bivalves as a consequence of shifting environmental conditions^20, 33^. Hence, as also confirmed by the present study, TEF of bivalve shells are not likely to remain stable over two consecutive years^27, 28, 33^.

In the present study, cockle shells from each ecosystem displayed contrasting TEF, the sole exception being those originating from Mira estuary (ME) and Ria de Alvor (RAl) (Table 1). Nevertheless, linear discriminant analysis (LDA) results showed that, over three-quarters (90%) of cockles were correctly classified to their areas (Table 2). This result highlights the need to combine different statistical tools (other than analysis of variance) when aiming to use biogeochemical signatures to assign a given specimen to a certain geographic origin. The results reported in the present study to successfully allocate a sampled specimen to its geographic origin are higher than those reported in previous studies addressing other bivalves (mussel species) along the coast of California (56%)^24^ and the Gulf of Maine (68%)^29^. Such an accurate identification of geographic origin as the one achieved for *Cerastoderma edule* in along the Portuguese coast is likely to be due to a combination of several factors, such as anthropogenic pressure (influencing Mn/Ca)^34^ and an increased input level of trace elements originating from terrestrial runoff (e.g. Mn/Ca and Ba/Ca)^35^ and their preferential retention in estuaries and coastal lagoons. It is also worth highlighting that the incorporation of trace elements may be significantly affected by shifting water temperature (affecting Mg/Ca and Sr/Ca)^36^ and salinity (affecting all ratios determined in this study)^37^, which due to the distinctive morphodynamics of each of the ecosystems surveyed may shift in a number of unique patterns and combinations. The higher ratios of Mg/Ca recorded in cockle shells from specimens in the northernmost locations surveyed (Ria de Aveiro (RAv) and Óbidos lagoon (OL)) are in accordance with the latitudinal temperature gradient displayed by this element in seawater^37, 38^. It is also worth highlighting that such differences may also be associated with more or less pronounced shifts in water temperature promoted by the size and shape of the estuarine systems surveyed (e.g. small and large estuaries and coastal lagoons).

The highest concentrations of Ba/Ca in TEF is often derived from freshwater inputs and nutrient runoffs to estuarine systems, which are known to promote an increase in primary productivity^35, 39, 40^. It is therefore possible that the runoffs originating from the fertile lands in the margins of RAv, OL and Ria Formosa (RF) that are used for agricultural purposes may contribute to the occurrence of diatom blooms that can increase the availability of Ba^35^. Concerning the significantly higher levels of Mn/Ca present in cockle shells originating from Tagus estuary (TE) it is likely associated with the legacy of former anthropogenic actions on the sampling site (namely historical metal industries), which has promoted the build-up of trace metals on surface water and sediment^34^.

Estuaries are dynamic environments in which coastal fluxes of trace elements and mixing rates with seawater vary from a tidal to an annual time scale^41^. In this way, it was not surprising to record significant shifts in the TEF of cockle shells of adult specimens originating from 4 different areas in RAv over two consecutive years. Indeed, Cathey *et al*.^33^ had already reported significant temporal variability (tri-weekly) in the TEF of larval bivalve shells being cultured in different hatcheries employing estuarine water in their operation in the southern Delmarva Peninsula (Virginia, USA). Carson^28^ also refers the occurrence of inter-seasonal and inter-annual variation in the TEF of Olympia oyster (*Ostrea lurida*) shells sampled in four different estuaries in Southern California (USA). Nonetheless, it is important to highlight that although larval and young juvenile TEF of bivalve shells may significantly shift in a weekly basis^24, 42^, older juveniles are known to display stable TEF over a six-month period^43^. Within the scope of using TEF of bivalve shells to verify claims on the geographic origin of adult live specimens traded for human consumption, temporal variability will likely not be an issue if samples to verify the claim can be collected from the same area of the specimens being traded. These TEF will certainly match, as the time scale of shelf-life for live bivalves is measured in days and not weeks or months. However, if this matching is performed using the TEF of bivalves originating from the same area but analyzed more than a year ago (e.g. by using data from a TEF database), temporal variability may significantly bias the analysis and an erroneous assignment of geographic origin is likely to occur. This pitfall associated with temporal variability is less likely to take place when comparing specimens originating from different ecosystems^28^.

Overall, the present study reinforces the potential that the TEF of cockle shells hold to be used as proxies for inferring the place of origin in traceability frameworks. Nonetheless, the analytical costs associated with the determination of TEF in bivalve shells whenever the need arises to verify claims related with their geographic origin can rapidly become prohibitive. In this scenario, it may be possible to rely on TEF previously recorded for specimens originating from the area being claimed by the producer/collector/trader. The time window during which this comparison can be performed without being prone to bias caused by temporal variability is likely to span between six months and less than a year. Future studies should try to determine, as accurately, as possible the span of such time window, as well as verifying if it does not change among capture/production areas.

## Material and Methods

### Samples collection and preparation

Cockles (*Cerastoderma edule*) were collected during low tide over June-July (Summer) 2014 on eight estuarine ecosystems along the Portuguese coast (Fig. 1): Ria de Aveiro (RAv) (Fig. 1a), Óbidos lagoon (OL) (Fig. 1b), Tagus estuary (TE), Albufeira lagoon (AL), Sado estuary (SE) (Fig. 1c), Mira estuary (ME) (Fig. 1d), Ria de Alvor (RAl) (Fig. 1e) and Ria Formosa (RF) (Fig. 1f). At the sampling moment, all locations were classified as “B” or “C” (Fig. 1) according to Council Regulation 853/2004 and 854/2004 of the European Union (EU)^12, 13^. In RAv four different areas were sampled and ten cockles collected per area (1 ecosystems × 4 areas × 10 replicates = 40 samples) (Fig. 1a). In OL, TE, AL, SE and RF two areas were sampled, with ten cockles also being collected on each one them (5 ecosystems X 2 areas X 10 replicates = 100 samples). As the bivalve species being surveyed was not abundant in ME and RAl only one area was sampled per ecosystem, with ten cockles being collected on each of them (2 ecosystem X 1 area X 10 replicates = 20 samples).

To evaluate the temporal variability of trace element fingerprints (TEF) in cockle shells, samples collected in the present study in RAv (Fig. 1a) were compared to those of specimens collected exactly in the same locations in the previous year (July (Summer) 2013). Ten specimens were sampled on each area in the two consecutive years (1 ecosystem × 4 areas × 2 years × 10 replicates = 80 samples). It is important to highlight that only cockles with approximately 3 years old (shell length 20–25 mm, commercial size)^44^ were sampled and that the time window for the present study (summer) matched that when the capture and trade of this bivalve species is higher and fraudulent practices are more likely to occur. All samples were collected by hand-raking, stored in aseptic food grade plastic bags and kept refrigerated during sampling. All specimens were frozen at −20 °C in the same day of collection for further analysis.

### Elemental analysis of cockle shells

Prior to elemental analysis of shells, all the polyethylene material, ceramic coated blades and tweezers were cleaned as described in detail by Ricardo *et al*.^20^. The valves were separated and the organic tissues were removed using ceramic coated blades and tweezers. The whole right valve was transferred to a previously acid-washed plastic bottle and the left valve discarded. Trace elements of the whole valve were obtained by the digestion method described by Ricardo, *et al*.^20^. Briefly, samples were soaked in 20 mL high-purity H_2_O_2_ (30% w/v) (AnalaR NORMAPUR, VWR Scientific Products) overnight (14–16 h) to remove organic matter from the shell including the periostracum. After organic matter removal Digestion of entire valves was performed with addition of 20 mL of high-purity concentrated (70% w/v) HNO_3_ (Trace metals; Sigma-Aldrich). To avoid having Ca masking the concentrations of the remaining elements^32, 45^, the resulting solution was diluted with Milli – Q (Millipore) water to a final acid concentration of 2% HNO_3_. Procedural blanks were prepared using the same analytical procedure and reagents of the samples. Barium (measured as ^137^Ba) and manganese (measured as ^55^Mn) present in *C*. *edule* shells were measured by inductively coupled plasma mass spectrometry (ICP-MS) on a Thermo ICP-MS X-Series equipped with an auto sampler CETAX ASX-510, Peltier Nebulizing Camera Burgener nebulizer, with nickel cones and the CeO+/Ce+ ratio being optimized at <2%. Calcium (measured as ^43^Ca), magnesium (measured as ^24^Mg) and strontium (measured as ^88^Sr) were determined through inductively coupled plasma optical emission spectrometry (ICP-OES) on a ICP-OES Jobin Yvon Activa M equipped with auto sampler JY-AS500 and Burgener Mira Mist nebulizer. The precision and accuracy of the analytical procedures were ensured by the analysis of certified reference materials (MRC’s) for sediments (Table S1 on Supplementary Information) while, the operating conditions are in Table S2 on Supplementary Information.

### Statistical analysis

The concentration of trace elements present in the shells of cockles was expressed as a ratio relatively to Ca^20, 26^, with these ratios always being calculated prior to any statistical analysis. A preliminary analysis of multivariance (MANOVA) was performed to detect significant differences (*p* < 0.05) in the TEF of shells from specimens originating from different areas within each ecosystem. As significant differences were recorded (Table S3 Supplementary Information), only the closest areas to the inlet from each ecosystem were selected for further analysis. The rationale supporting this decision was the assumption that the areas closer to the inlet of each ecosystem are more similar among them due to the environmental influence of ocean conditions and, consequently, these locations would likely be more challenging to discriminate TEF from cockle shells. A total of 10 replicates per ecosystem were therefore used for further statistical analysis. In order to determine whether there were any significant differences (*p* < 0.05) in the TEF of cockle shells among ecosystems an analysis of multivariance (MANOVA) was performed. As significant differences were recorded, one-way analysis of variance (ANOVA) were performed for each elemental ratio. Whenever the ANOVA revealed the existence of significant differences (*p* < 0.05) a post hoc test (Tukey’s test) was performed to identify which ecosystems differed from each other. A linear discriminant analysis (LDA) was performed to assess the reliability of using TEF displayed by cockle shells to infer their geographic origin.

A MANOVA was used to evaluate the inter-annual stability of TEF displayed by cockle shells over two consecutive years (2013 and 2014) within the four sampled areas of RAv. Due to the significant differences recorded, an ANOVA was applied (as detailed above), with post hoc tests (Tukey’s test) being employed when applicable. All analyses were performed on log X + 1 transformed data, in order to meet the normality and homogeneity of variance of ANOVA and the multivariate normality and homoscedasticity (Pillai Trace test) are required for MANOVA. All statistical analysis were performed using R^46^.

## Electronic supplementary material

Suppl. Inf.
