# Supplementary material for: Spatio-temporal variability of trace elements fingerprints in cockle (Cerastoderma edule) shells and its relevance for tracing geographic origin
Source: Sci Rep. 2017 Jun 14;7:3475. doi: 10.1038/s41598-017-03381-w (PMC5471190; doi:10.1038/s41598-017-03381-w)
Supplement: Supplementary file 1 — Suppl. Inf. [file 41598_2017_3381_MOESM1_ESM.pdf]

Supplementary information

Spatio-temporal variability of trace elements fingerprints in cockle (*Cerastoderma edule*) shells and its relevance for tracing geographic origin

Fernando Ricardo*, Tânia Pimentel, Luciana Génio and Ricardo Calado*

Departamento de Biologia & CESAM, Universidade de Aveiro, Campus Universitário de Santiago, 3810-193 Aveiro, Portugal

Corresponding authors:
*Fernando Ricardo (fernandoafricardo@gmail.com) and Ricardo Calado (rjcalado@hotmail.com)

Table S1. Detection limits (DL), percentages of samples above DL and precision estimates (% relative standard deviation, RSD) for the Inductively Coupled Plasma-Mass Spectrometer (ICP-MS) analysis of *Cerastoderma edule* shells. DL are based on blank analyses and are expressed in mmol ratios relative to Ca. External precision estimates are based on % RSD standards certified reference materials (MRC's) for sediments.

Element	DL (mmol^{-1} Ca)	% above DL	% RSD
Mg	0.619	100	3
Sr	1.885	100	3
Ba	0.002	100	3
Mn	0.009	100	2

Table S2. Inductively Coupled Plasma-Mass Spectrometer (ICP-MS) operating conditions

**Data acquisition parameters**	
Scanning	Peak jump
Dwell time (ms)	10
Reading per replicate	3
Points per spectral peak	1
Sweeps	60
**Setup timings**	
Uptake	30 s
Washout	60 s
**Requirements of argon gas**	
Purity	≥ 99,996%
Maximum quantity of water	<5 mg L^{-1}
Flow	14 L min^{-1}
**Isotopes measured**	^{24}Mg, ^{43}Ca, ^{88}Sr, ^{37}Ba, ^{55}Mn
**Internal standard**	^{115}In

Table S3. Multivariate analysis of variance (MANOVA) of the trace elements fingerprinting (TEF) of the *Cerastoderma edule* shells between areas within Óbidos Lagoon (OL), Tagus Estuary (TE), Albufeira Lagoon (AL) and Sado Estuary (SE).

Area	df	pillai	approx. F	p. value
OL1 vs OL2	1	0.910	38.10	1.08e-07
TE1 vs TE2	1	0.747	11.10	2.19e-04
AL1 vs AL2	1	0.444	3.00	5.25e-02
SE1 vs SE2	1	0.459	2.97	5.71e-02
RF1 vs RF2	1	0.831	18.50	1.15e-05
